# A molecular overlayer with the Fibonacci square grid structure

**DOI:** 10.1038/s41467-018-05950-7

**Published:** 2018-08-24

**Authors:** Sam Coates, Joseph A. Smerdon, Ronan McGrath, Hem Raj Sharma

**Affiliations:** 1 Surface Science Research Centre and Department of Physics, University of Liverpool, Liverpool, L69 3BX UK; 2 Jeremiah Horrocks Institute for Mathematics, Physics and Astronomy, University of Central Lancashire, Preston, PR1 2HE UK

## Abstract

Quasicrystals differ from conventional crystals and amorphous materials in that they possess long-range order without periodicity. They exhibit orders of rotational symmetry which are forbidden in periodic crystals, such as five-, ten-, and twelve-fold, and their structures can be described with complex aperiodic tilings such as Penrose tilings and Stampfli–Gaehler tilings. Previous theoretical work explored the structure and properties of a hypothetical four-fold symmetric quasicrystal—the so-called Fibonacci square grid. Here, we show an experimental realisation of the Fibonacci square grid structure in a molecular overlayer. Scanning tunnelling microscopy reveals that fullerenes (C_60_) deposited on the two-fold surface of an icosahedral Al–Pd–Mn quasicrystal selectively adsorb atop Mn atoms, forming a Fibonacci square grid. The site-specific adsorption behaviour offers the potential to generate relatively simple quasicrystalline overlayer structures with tunable physical properties and demonstrates the use of molecules as a surface chemical probe to identify atomic species on similar metallic alloy surfaces.

## Abstract (web summary)

Quasicrystals possess long range order but no translational symmetry, and rotational symmetries that are forbidden in periodic crystals. Here, a fullerene overlayer deposited on a surface of an icosahedral intermetallic quasicrystal achieves a Fibonacci square grid structure, by selective adsorption at specific sites.

## Introduction

**Fig. 1:**
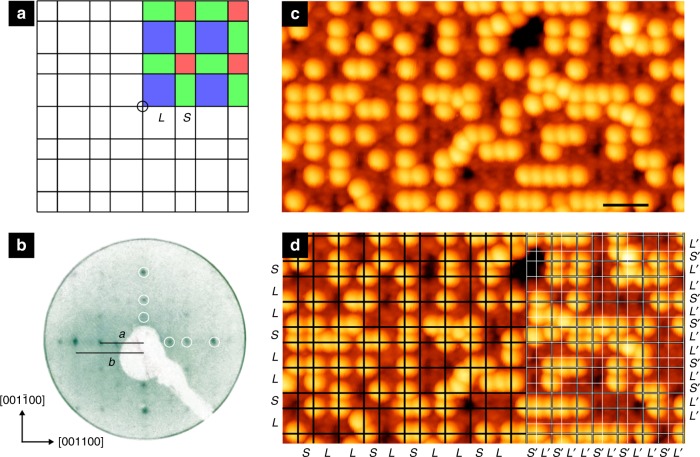
Fibonacci square grid, LEED and STM. **a** The Fibonacci square grid, with highlighted constituent tiles (blue: *L* × *L*, red: *S* × *S*, and green: *S* × *L*). The centre of rotation of four-fold symmetry is marked by a circle. **b** Low-energy electron diffraction pattern (60 eV, inverted for clarity) from the clean two-fold surface of the icosahedral (*i*) Al–Pd–Mn quasicrystal. Diffraction spots are *τ*-scaled in the primary two-fold axes (highlighted with white circles), with two reciprocal space lengths indicated: *a* = (14.1 ± 0.3) nm^−1^ and *b* = (22.5 ± 0.3) nm^−1^. **c** Quasicrystalline C_60_ on the two-fold *i*-Al–Pd–Mn quasicrystal as imaged by scanning tunnelling microscopy. Scale bar represents 4 nm. **d** Image **c** overlaid with a Fibonacci square grid of *S* = 1.26 nm, *L* = 2.04 nm (black lines) to highlight the ordering of C_60_. A *τ*-deflated Fibonacci square grid of *L*′ = *L/τ* = *S* and *S*′ = *S/τ* is shown by white lines on the right-hand side. Note that the original grid overlaps with the *τ*-deflated grid

Quasicrystalline phases have been observed in a range of materials, including intermetallics^1^, liquid crystals^2^, polymers^3,4^, colloids^5,6^, perovskites^7^ and overlayer structures of single elements^8–11^ and molecules^12,13^. All of these quasicrystalline bulk and epitaxial phases exhibit forbidden rotational symmetries. In 2002 Lifshitz^14^ pointed out that quasicrystals are not exclusively defined in terms of possessing forbidden symmetries. He introduced the Fibonacci square grid, which exhibits four-fold symmetry, but is quasicrystalline^14^. The square grid is constructed by superimposing two orthogonal Fibonacci linear grids (Fig. 1a). The Fibonacci linear grid is produced using short (*S*) and long (*L*) sections and certain substitution rules: *S* → *L* and *L* → *LS*. These conditions create a sequence: *S*, *L*, *LS*, *LSL*, *LSLLS*… . When *L* = *τS*, where *τ* is the golden mean (${\textstyle{{1 + \sqrt 5 } \over 2}}$ = 1.618…), this sequence models a one-dimensional quasicrystal. The Fibonacci square grid is constituted by three tiles (*S* × *S*, *S* × *L* and *L* × *L*) highlighted in Fig. 1a by different colours. Like other complex aperiodic tilings, the Fibonacci square grid follows specific rules for tile placement and frequency, and exhibits *τ*-inflation symmetry^14^, i.e., the enlargement or shrinking of dimensional quantities by *τ*. The theoretical construct was later extended to a cubic Fibonacci tiling in three dimensions^15^. Lifshitz^14^ noted at the time that to the best of his knowledge, no alloys or real quasicrystals existed with the structure of the square or cubic Fibonacci tilings.

The physical properties of a hypothetical quasicrystal with the square Fibonacci grid structure have been explored theoretically, including its electronic, phononic and transport behaviours^15–17^. Dallapiccola et al.^18^ examined the plasmonic properties of a lithographically fabricated Fibonacci square grid of a sub-micrometre scale. Similarly, Vardeny et al.^19^ studied the photonic properties of a system with a stacking of slabs of two different periodic materials in a Fibonacci sequence, as an example of one-dimensional quasicrystalline structure. In this work we present experimental observation of the Fibonacci square grid in a molecular overlayer system.

## Results

### Substrate structure

We used the surface of an intermetallic quasicrystal, icosahedral (*i*)-Al–Pd–Mn, as a template for C_60_ adsorption. The icosahedral quasicrystal possesses two-, three- and five-fold rotational symmetry axes. Here we have used a surface perpendicular to the two-fold axis. The substrate surface was prepared as explained in the Methods section. Low-energy electron diffraction (LEED) and scanning tunnelling microscopy (STM) were utilised to characterise the surface.

**Fig. 2:**
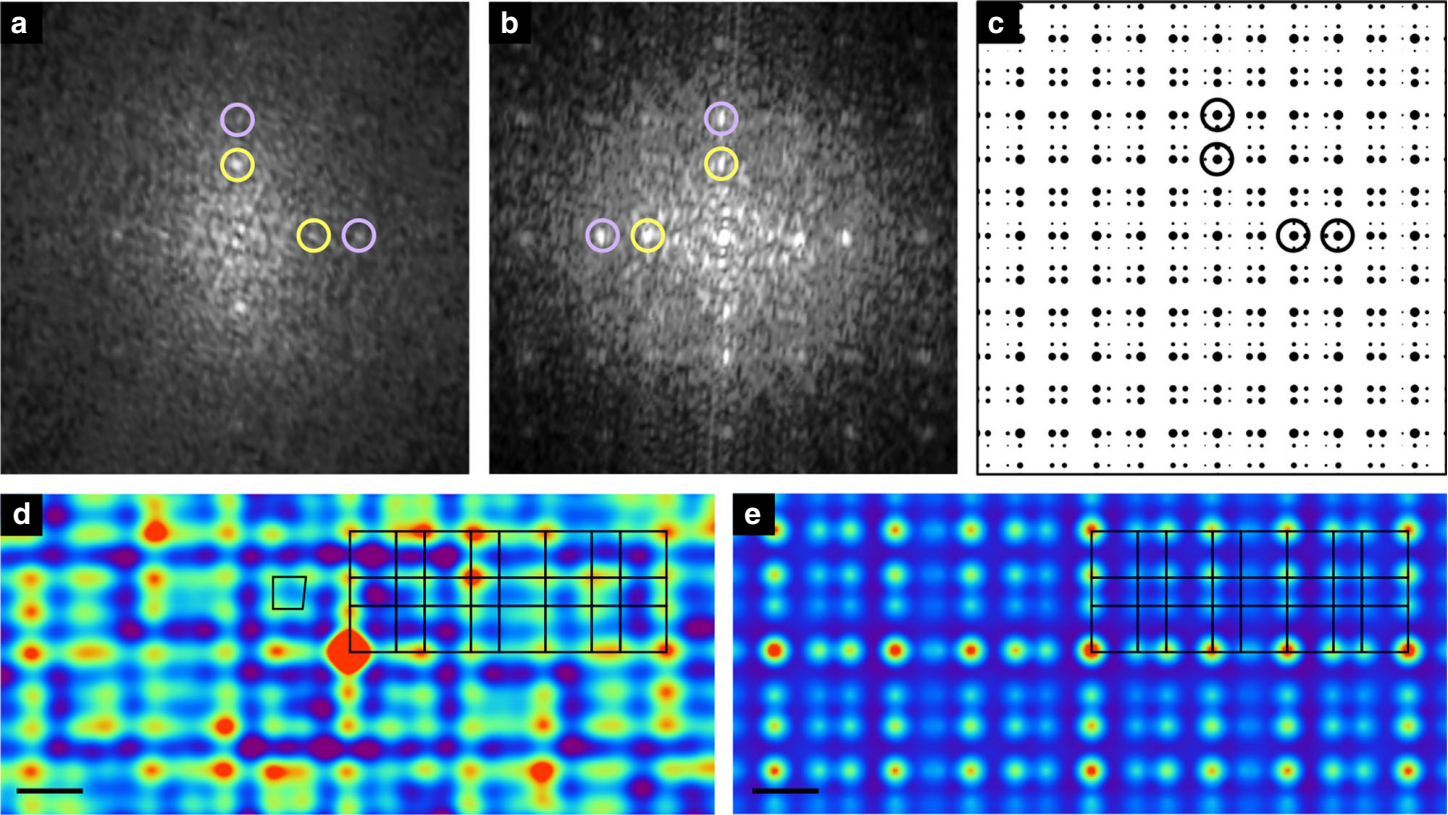
Fast Fourier transform and autocorrelation function. **a** Fast Fourier transform (FFT) of a scanning tunnelling microscopy (STM) image of the clean surface, demonstrating two-fold symmetry. **b** FFT of the C_60_ molecules of Fig. 1c. **c** FFT generated from a Fibonacci square grid, showing four-fold symmetry. Spots of the first two orders of diffraction are highlighted by circles of different colours. FFTs in **a**, **b** are displayed with the same *k*-vector scale, while the scale of **c** is arbitrary. **d** Autocorrelation function of the STM image of the C_60_ overlayer of Fig. 1c. A distorted square is marked. **e** Autocorrelation function taken from point objects at the vertices of a Fibonacci square grid of *S* = 1.26 nm, *L* = 2.04 nm. Scale bars in **d**, **e** represent 1 nm

The analysis of *k*-vectors of the LEED pattern (Fig. 1b) reveals that the surface corresponds to a bulk termination (Supplementary Note 1). This means that the surface has two-fold symmetry, as expected from the bulk. Consistent with the LEED results, fast Fourier transforms (FFTs) of STM images from the clean surface are also two-fold (Fig. 2a, see further discussion later). STM images can also be explained as bulk atomic planes. The observation of two-fold symmetry of the surface is in agreement with previous STM results^20,21^ (Supplementary Note 2).

### STM of C_60_ overlayer

When C_60_ is deposited on the clean surface at 600 K, a quasicrystalline network is formed, as shown in Fig. 1c. A majority of the C_60_ molecules (70% of the total observed) lie at the vertices of a Fibonacci square grid of *S* = 1.26 nm, *L* = 2.04 nm. This grid is superimposed in Fig. 1d (black lines). The remaining minority of C_60_ can be placed at the vertices of a *τ*-deflated grid. The *τ*-deflated grid is shown by white lines in Fig. 1d, where *L*′ = *L*/*τ* = *S* and *S*′ = *S*/*τ*. The origin of low occupancy of C_60_ at the *τ*-deflated vertices will be discussed later.

We present FFT patterns taken from the clean and C_60_ dosed surfaces in Fig. 2a, b. For the calculation of the C_60_ pattern, the substrate was filtered out so that only the molecular film made a contribution to the FFT. Spots with equal *k*-vectors are highlighted by circles, indicating first- and second-order spots. The FFT spots for the substrate (Fig. 2a) and C_60_ (Fig. 2b) appear at the same *k*-vectors but their intensity distribution is different. The real space values corresponding to the *k*-vectors of FFTs are related to the length scales of *L* and *S*.

The Fibonacci square grid structure of the C_60_ overlayer is confirmed by comparing the FFT of the STM image with the reciprocal space transform of the Fibonacci square grid (Fig. 2c). A grid of *S* (=1.26 nm) and *L* (=2.04 nm) lengths was chosen to allow for a direct comparison to the experimental FFT results. The pattern fits extremely well with the FFT of the C_60_ overlayer (Fig. 2c).

We also compare autocorrelation functions that are calculated from the STM image and from a model Fibonacci square grid. Figure 2d is the autocorrelation function of the C_60_ molecules in the STM image shown in Fig. 1c. Similar to the FFT of the C_60_ molecules (Fig. 2b), the contribution from the substrate in STM was removed so that the autocorrelation function arises solely from the C_60_ molecules. Figure 2e shows the autocorrelation function of a perfect grid, i.e., a point object is placed at every vertex of a Fibonacci square grid of *S* = 1.26 nm and *L* = 2.04 nm. The size of grid used to calculate the autocorrelation was 50 nm × 50 nm, but we have shown only a section for comparison. As expected, spots in the autocorrelation function of the model grid form a perfect Fibonacci square grid. In agreement with the model, the autocorrelation function of the STM image can also be mapped by a Fibonacci square grid. The tile lengths of the grid are also *S* and *L*. This means that C_60_ molecules at the *τ*-deflated grid do not contribute to the autocorrelation pattern. This is expected, as only a fraction of the *τ*-deflated vertices are occupied by C_60_. In a perfect Fibonacci square grid, the number of vertices in the *τ*-deflated grid is *τ*^2^ times the number of vertices in the original grid. The relative density of vertices of the *τ*-deflated grid that do not overlap with the original grid is thus *τ*^2^ − 1 = *τ*, i.e., there are ~162% more than the vertices of the original grid. However, only ~8% of these vertices are occupied by C_60_ as observed by STM, and therefore these molecules do not produce additional features in the autocorrelation pattern.

## Discussion

**Fig. 3:**
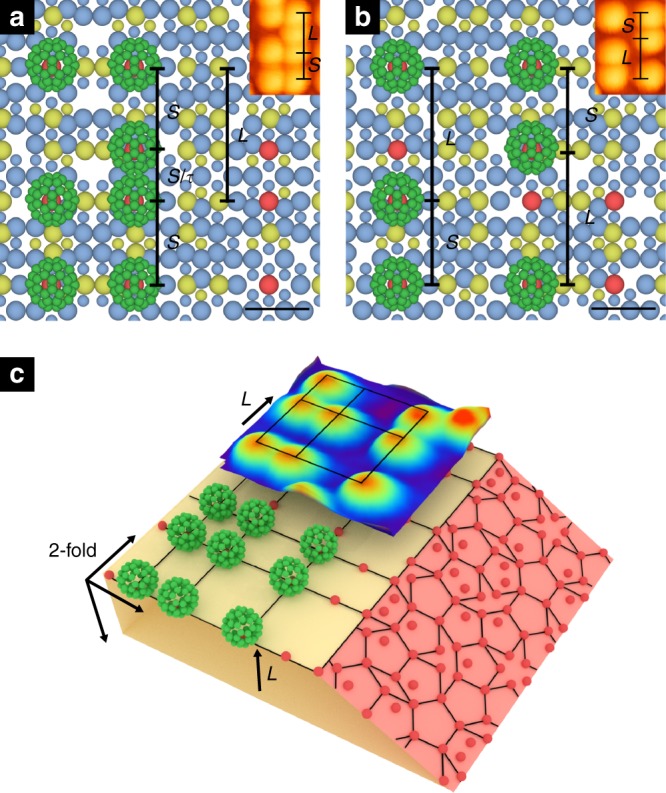
Adsorption sites. **a**, **b** Comparison between C_60_ motifs observed by scanning tunnelling microscopy (STM) and the model structure of icosahedral (*i*)-Al–Pd–Mn. Model structures showing C_60_ adsorbed atop Mn atoms, forming *S* × *S* and *S* × *L* tiles. Two images are taken from two different sections of the surface plane in order to illustrate common STM features. The insets in **a**, **b** are sections of the STM image from Fig. 1c. The inset in **a** shows an additional C_60_ adsorbed at a *τ*-deflated position, which appears as a squashed (i.e., non-circular) molecule. Al = blue, Pd = yellow, Mn = red and C_60_ = green. Atoms in different layers are presented in different sizes, with the largest in the top layer. Scale bars in **a**, **b** represent 1 nm. **c** A block of the model structure of *i*-Al–Pd–Mn displaying five-fold (pink) and two-fold (cream) planes, with C_60_ adsorbed atop Mn atoms. The orientation of C_60_ is arbitrary. Only Mn atoms are shown for clarity. There are three equivalent two-fold axes in icosahedral quasicrystal, which are orthogonal to each other: two of them are in the surface plane and the third is perpendicular to the surface plane. These directions are indicated by arrows. An STM image is superimposed for comparison

The specific adsorption sites of the molecules will now be considered. For this, we present the atomic structure of the two-fold surface of the *i*-Al–Pd–Mn quasicrystal in Fig. 3a, b. The structure corresponds to high-density planes of the bulk atomic model proposed by Boudard et al.^22^. The STM images of the substrate are consistent with these atomic planes (see Supplementary Note 2). Following direct measurement from the STM data (Fig. 1c) and comparison with theoretical autocorrelation results (Fig. 2e), the size of the C_60_ Fibonacci grid is known. This grid (*S* = 1.26 nm, *L* = 2.04 nm) fits with the separations of the Mn atoms in the surface plane (Fig. 3a, b), suggesting that individual C_60_ molecules adsorb directly on top of these Mn atoms. Aluminium adsorption sites are ruled out, as although Al atoms are separated by similar *S* and *L* lengths, the Al density at the surface would provide too many adsorption sites—producing a disordered film. This is also true for Pd sites.

The Mn-based adsorption model is further strengthened by the Fibonacci square grid structure of the C_60_ overlayer and the substrate model structure. We present a block of the atomic structure of the *i*-Al–Pd–Mn quasicrystal, simultaneously displaying the five- and two-fold planes (Fig. 3c). For clarity we have shown only Mn atoms in the model. Mn atoms are located at the centre of pseudo-Mackay clusters, the building blocks of the *i*-Al–Pd–Mn quasicrystal^23^. The five-fold surface intersects the centre of the clusters and the Mn atoms can be mapped with a Penrose P1 tiling. The edge length of the tiling is ~0.77 nm, which is confirmed by STM^24^. The two-fold surface terminates at specific planes of the P1 tiling, such that Mn atoms form a Fibonacci square grid. A few remaining Mn atoms are located at *τ*-deflated positions. Two of these planes are marked in Fig. 3c. However, Al and Pd atoms at the surface plane do not form a Fibonacci square grid. They show two-fold symmetry.

A comparison between the detailed structure of the C_60_ overlayer observed by STM, and Mn atomic positions in the model structure, also indicates that Mn atoms are the bonding sites. Figure 3a shows *S* × *S* and *S* × *L* tiles from the model whose vertices are decorated with C_60_. An additional C_60_ molecule occupies at an *S*/*τ* (0.77 nm) deflated position. The inset on Fig. 3a is an STM image of the corresponding motif. In the model, the *S*/*τ* Mn position is too close to its nearest Mn neighbour to allow neighbouring C_60_ molecules to adsorb without significant molecule–molecule interaction. The steric interaction between neighbouring C_60_ at these positions thus results in a slight spatial displacement away from the Mn adsorption sites. This displacement translates into the autocorrelation function, where some spots are displaced from the perfect Fibonacci grid producing deformed squares, see a marked distorted square in Fig. 2d. However, these *τ*-deflated positions can also be occupied without C_60_–C_60_ displacement, as Fig. 3b shows. Here, C_60_ molecules occupy Mn sites that create inverted Fibonacci sequences (again, inset is an STM motif). These positions may also contribute to the deformation of the experimental autocorrelation spots, as they simultaneously represent two inverted square grid tiles (*S* × *S* and *S* × *L*). In addition, the density of Mn atoms at *τ*-deflated positions is very low in the model structure, compared to their density at the original grid. This is consistent with the low density of C_60_ at the *τ*-deflated grid.

C_60_ is an excellent electron acceptor^25^ and Mn is electron rich^12^. Therefore, a strong electronic molecule–substrate interaction at Mn sites is expected. Such behaviour was previously observed for Bi and Si on the five-fold surface of *i*-Al–Pd–Mn, where the adsorbates were observed to bond to Mn atoms^9,26^. Similarly, C_60_ was found to bond to Fe on the five-fold *i*-Al–Cu–Fe surface^13^. The *i*-Al–Cu–Fe quasicrystal and *i*-Al–Pd–Mn have a very similar structure^27^.

In conclusion, we have shown that C_60_ molecules form a Fibonacci square grid on the two-fold surface of *i*-Al–Pd–Mn. The observation of such a structure extends the quasicrystal family beyond the forbidden symmetry systems previously observed. More generally, the sparse density of the minority constituents of complex metallic alloys presents a unique adsorption landscape for the construction of molecular arrangements on a chosen scale; for example, this methodology could permit the construction of a molecular magnet array of chosen magnetic behaviour. Additionally, understanding the propagation of waves inside five-, ten-, and twelve-fold quasicrystalline lattices as waveguides is an attractive problem due to the nearly isotropic Brillouin zones. However, it is a rather intractable one. A suitably constructed simplified Fibonacci square grid structure could therefore provide a useful stepping-stone to the understanding of complex phenomena such as the formation of photonic quasicrystals^19^. We have also shown that C_60_ can be utilised as a chemical probe that can tag unique adsorption sites of a complex structure, and thus provide an insight into the surface structure. This technique can of course be extended to other quasicrystalline and complex metallic alloy surface structures, providing that the experimental conditions are adequate; primarily the surface must have a unique or sparse enough adsorption network to provide meaningful topographic data.

## Methods

### Surface and thin film preparation

A two-fold Al–Pd–Mn quasicrystal was polished with successively finer grades of diamond paste from 6 μm down to 0.25 μm, before solvent washing with methanol in an ultrasonic bath. Upon insertion into ultra-high vacuum the sample was cleaned by several sputter–anneal cycles (30 min sputtering, 2 h anneal at 900 K) to form a flat surface. Surface ordering and cleanliness was assessed with LEED. STM was used to investigate local atomic and molecular arrangements. C_60_ was deposited using thermal evaporation, while the substrate was held at 600 K. The surface was exposed to the source at a rate of 0.1 monolayer per minute. A range of molecular coverages were investigated.

### Data availability

The main data supporting the findings of this study are included in this article and its Supplementary information files. Additional STM data are available from the corresponding author upon request.

## Electronic supplementary material

Supplementary Information
