# Supplementary material for: A molecular overlayer with the Fibonacci square grid structure
Source: Nat Commun. 2018 Aug 24;9:3435. doi: 10.1038/s41467-018-05950-7 (PMC6109137; doi:10.1038/s41467-018-05950-7)
Supplement: Supplementary file 1 — Supplementary Information [file 41467_2018_5950_MOESM1_ESM.pdf]

A molecular overlayer with the Fibonacci square grid structure: supplementary information

Sam Coates[1], Joseph A. Smerdon[2], Ronan McGrath[1] & Hem Raj Sharma[1]

[1]*Surface Science Research Centre and Department of Physics, University of Liverpool, Liverpool L69 3BX, UK*

[2]*Jeremiah Horrocks Institute for Mathematics, Physics and Astronomy, University of Central Lancashire, Preston, PR1 2HE, UK*

Supplementary Note 1: LEED from the clean 2-fold surface of *i*-Al-Pd-Mn

A LEED pattern from the clean surface is shown in Figure 1b of the main text. Two reciprocal space lengths indicated are *a*=(1.41±0.03) Å^{-1} and *b*=(2.25±0.03) Å^{-1}. The reciprocal space vectors were determined by comparing LEED patterns from the Cu(111) surface at the same energy. Quasiperiodic ordering is indicated by the ratio of *b*/*a*=(1.59±0.04), which agrees within experimental error with the numerical value of τ. Icosahedral quasicrystal diffraction patterns are indexed using 6 bulk reciprocal space basis vectors[1]. By projecting these vectors onto the surface considering the relevant rotational orientation (i.e. 2–, 3–, or 5–fold), the surface induced LEED spots can be indexed with respect to the bulk. The surface–projected vector along the [001100] direction has a length of 1.45 Å^{-1}[1]. There is a good agreement between this value and *a* (±3%), which indicates that the surface is consistent with a bulk termination.

Supplementary Note 2: STM from the clean 2-fold surface of *i*-Al-Pd-Mn

STM reveals a step-terrace structure of the surface, in agreement with previous studies[2,3]. The terraces appear rough, with the average root mean square roughness = 0.046±0.004 nm. They show three distinct features (Supplementary Figure 1a,b): bright protrusions, patches of darker contrast and the remaining row-like features with intermediate contrast, which run top to bottom of the image. The protrusions line up along the orthogonal direction of the rows, i.e., the horizontal direction in the image. The orientation of the rows and protrusions reflects the 2-fold symmetry of the surface. The rows are separated by distances S=1.26 nm or L=2.04 nm = $S \times \tau$, and these spacings form a Fibonacci set. No such Fibonacci sequence is observed in the orthogonal direction. However, features along this direction have characteristic lengths, which are related to S by $S\tau^n$ (n an integer) (see features marked by rectangles in Supplementary Figure 1a). The observed results are in good agreement with previous STM studies on the same surface by Reid *et al.*[2] and Gröning *et al.*[3]. These papers also reported STM images with dark patches, protrusions and row-like features. STM images were found to exhibit characteristic lengths, identical to S, or τ-scaled to S. For example, Reid *et al.* shows a 2-fold Fibonacci grid of length scale similar to S and L[2]. Gröning *et al.* reported rectangular features, which have the same size or τ-scaled of those marked in Supplementary Figure 1a[3]. In agreement with our current observations, the surface exhibited 2-fold symmetry, as expected from the bulk.

The terrace structure can be explained by a combination of two closely separated atomic planes of the bulk, whose collective density is equivalent to a single dense plane. The surface planes

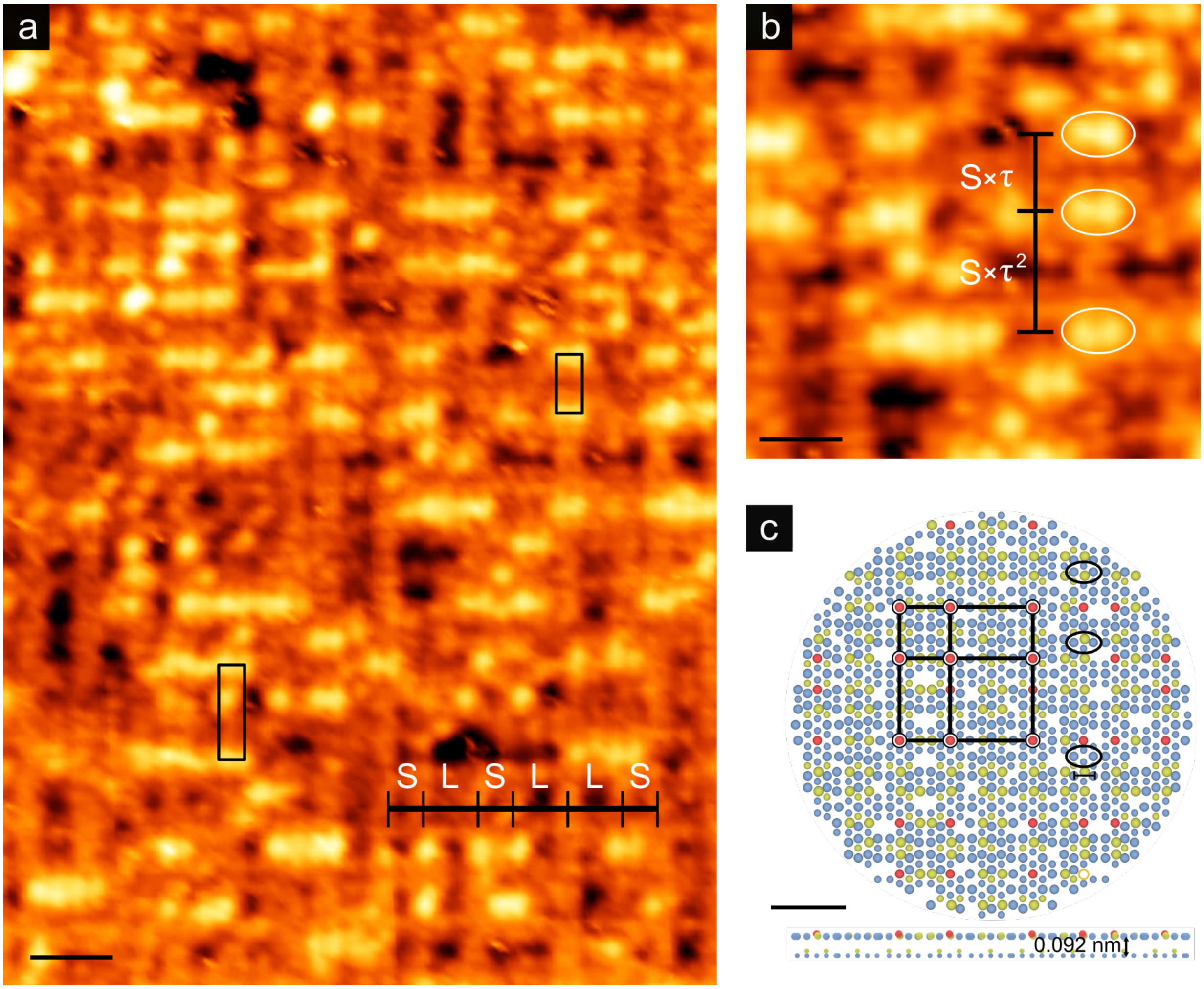

Supplementary Figure 1: **Clean surface.** (**a**) STM of the clean 2–fold Al–Pd–Mn surface. (**b**) Magnified view of a section of image (**a**), with highlighted bright ‘dimer’ protrusions. (**c**) Model structure of the 2–fold Al–Pd–Mn surface, where Al = blue, Pd = yellow and Mn = red. Atomic layers in a slab of 0.092 nm thickness are considered. Atoms in different layers are shown with different sizes, the largest being in the top layer. Highlighted is a Fibonacci square grid, and Al atoms responsible for the bright protrusions in **a**. The corresponding structure in a side view is shown at the bottom. Scale bars in (**a**), (**b**) and (**c**) represent 3, 2 and 2 nm, respectively.

selected are shown in Supplementary Figure 1c. The two planes have a separation of z=0.092 nm. As we describe below, the protrusions in STM can be matched to features in the incomplete top layer of the model structure and the row-like features are related to the lower plane, while the darker contrasts are vacancies due to atoms desorbed from the first and second atomic planes.

The protrusions most often appear as dimers, i.e., two bright spots separated by 0.49±0.02 nm (highlighted in Supplementary Figure 1b). This value agrees with the inter–atomic distance of Al atoms in the top layer of the model, 0.484 nm (Supplementary Figure 1b). The separation of the dimers in STM is S=1.26$\times\tau^n$ nm (n=1, 2, 3...). In the model the minimum distance of these dimers is 0.780 nm in x and 0.296 nm in y, which is related to the S value measured in Supplementary Figure 1b by τ^n deflation. Thus, the distribution of dimers in the model can model Fibonacci sequences of the particular length scale observed by STM. The protrusions have an average height above the mean substrate of 0.089±0.004 nm, which is similar to the separation of the top two layers, 0.092 nm. The row-like features of Supplementary Figure 1a can be identified as Al-dense rows formed in the bottom surface plane, upon which the Al dimers of the top layer sit. This is concluded based on the separation of the rows and their relative location with the dimers in STM and model. Density functional theory calculations performed by Krajčí *et al.*[4] show that high concentrations of Al/Mn atoms in quasicrystals are often observed with bright contrast by STM. Therefore, this calculation supports the argument that the brighter features observed by STM are related to Al atoms. The depth of the darkest features in STM is 0.25±0.2 nm below the bright protrusions. The next atomic plane below the selected surface termination in the model lies 0.24 nm below the top layer. This suggests that the darkest features are vacancies created by desorbing

atoms from the top layers. We note that other systems, such as an approximant of Al-Pd-Mn quasicrystal[5], also show such incomplete top layers.

Supplementary references

1. Sharma, H. R., Shimoda, M. & Tsai, A. P. Quasicrystal surfaces: structure and growth of atomic overlayers. *Advances in Physics* **56**, 403–464 (2007).

2. Reid, D., Smerdon, J. A., Ledieu, J. & McGrath, R. The clean and copper-dosed two-fold surface of the icosahedral Al-Pd-Mn quasicrystal. *Surface Science* **600**, 4132–4136 (2006).

3. Gröning, O., Widmer, R., Ruffieux, P. & Gröning, P. Scanning tunnelling microscopy with atomic resolution on the twofold surface of the icosahedral AlPdMn quasicrystal. *Philosophical Magazine* **86**, 773779 (2006).

4. Krajčí, M., Hafner, J., Ledieu, J. & McGrath, R. Surface vacancies at the fivefold icosahedral Al-Pd-Mn quasicrystal surface: A comparison of ab initio calculated and experimental STM images. *Physical Review. B* **73**, 024202 (2006).

5. Fournée, V. *et al.* Surface structures of approximant phases in the Al-Pd-Mn system. *Physical Review B* **66**, 165423 (2002).
